# Roughness Control of Surfaces Using a Laser Profilometer with the Selected Material Cutting Technology

**DOI:** 10.3390/ma16114109

**Published:** 2023-05-31

**Authors:** Juraj Ružbarský

**Affiliations:** Faculty of Manufacturing Technologies, Technical University of Kosice, Sturova 31, 080 01 Presov, Slovakia; juraj.ruzbarsky@tuke.sk

**Keywords:** quality, cutting, profilometer, roughness, parameter

## Abstract

The article aims to assess the roughness of parting surfaces in the context of abrasive water jet technology for various materials. The evaluation is based on the feed speed of the cutting head, which is adjusted to achieve the desired final roughness, taking into consideration the stiffness of the material being cut. We used non-contact and contact methods to measure selected parameters of the roughness of the dividing surfaces. The study included two materials—namely, structural steel material S235JRG1 and aluminum alloy AW 5754. In addition to the above, the study involved using a cutting head with varying feed rates to achieve different surface roughness levels required by customers. The roughness parameters Ra and Rz of the cut surfaces were measured using a laser profilometer (laser profilometer). To ensure the accuracy of the laser profilometer, a control roughness measurement was conducted using a contact roughness gauge. The roughness values obtained for Ra and Rz from both measurement methods were plotted on a graph to illustrate their dependencies and were subsequently evaluated and compared. By measuring the roughness parameters Ra and Rz, the study was able to provide insights into the effectiveness of the cutting head’s feed rates in achieving the desired roughness levels. Additionally, by comparing the results of the laser profilometer and contact roughness gauge, the accuracy of the measurement non-contact method used in the study was verified.

## 1. Introduction

The quality of machined surfaces is constantly growing in importance and becoming increasingly demanding. Surface quality has a major impact on the functionality of the entire equipment [1].

Fine micro geometric surface irregularities deviating from the ideal surface are referred to as surface roughness, caused by the tool used in machining and finishing [2].

The surface texture of the material, which can take many forms (i.e., fine grooves, grooved structures created by machining, etc.), is gaining importance, especially in the field of surface engineering [3].

The parameter of roughness is one of many factors that define the quality of machining, the effect of which is increased productivity. In terms of mechanical vibrations, higher values of roughness are undesirable because they cause noise and dynamic stress. These result in fatigue, malfunction of the mechanical structure or its functional parts, loss of energy, or loss of performance [4,5].

Experimental analysis of surface roughness in the process of machining is important in several ways. Unwanted surface roughness may indicate damage to the functional parts of the machine tool, wear of the tools, the workpiece, the cutting head, or other parts of the machine tool. These processes then initiate damage to the machine’s structure, machine stiffness, bearing components, or other machining and cutting parts of the machine [6].

By evaluating the roughness parameters, it is possible to predict the sequence of operations necessary to achieve the required surface quality and also to optimize the process of its formation [7].

The value of surface roughness is often a critical factor for products that come into contact with each other [8]. It also has a significant impact on the durability, reliability, and operation of technical equipment [9,10].

The emerging surface roughness is not only a carrier of partial information, but above all, an image of the technology used to create it [11].

With the development of science, technology, and the application of the results of this development, the importance of surface quality of components also increases, with their durability and reliability largely affected [12,13].

Surface roughness is a technical value that has a critical influence on friction in contact surfaces. The surface roughness is assessed according to the size of protrusions and depressions in the surface. At present, contact profilometers and profile graphs are mainly used for the quantitative evaluation of the surface structure. Recently, however, new methods have emerged that bring several benefits. These digital instruments analyze the surface very precisely and evaluate the surface roughness parameters based on the data obtained. These are determined by optical vision, magnifying glass, microscope, light interference, or electrical measurement [2].

One of the most recently developed approaches to measuring surface roughness is 3D profilometry using a laser, while pulsed thermography has been used to combine the effect of surface roughness with thermal radiometry [14].

Contactless measurement methods replace contact measurements of surfaces sensitive to mechanical damage, soft materials, etc. Contactless measurement avoids damaging the measured surface. The inspected surface is monitored by a focused measuring head, whose program-controlled adjustment is quick and easy [2].

The setup of most experimental assemblies involving optical 3D methods is very similar in principle. There is always an optical (light) source, a measured object, and an optical detector in the assembly. Light sources are projected via the so-called optical probe, which is an optical structure, falling on the surface of the object measured. Individual methods differ from each other in the type of this optical structure and in the way of evaluating its interaction with the measured surface [12].

Optical profilometric systems are described as systems for real-time image reading, digital signal processing, and full-color 3D profile display. Ongoing discussions address the relevant operating principles, strengths, and weaknesses of these devices, as well as the main constraints and future challenges in the field of high-speed optical profilometry [15,16].

Optical measuring devices in which the scanning tip is replaced by an electromagnetic radiation beam avoid damaging the surface of the measured part. Radiation reaching the surface can be reflected in three different ways: reflection, scattering, or a combination of the two. The interaction between the radiation and the measured sample depends primarily on the surface roughness and the radiation wavelength used [2,15].

The main advantages of these devices are contactlessness, non-destructiveness, the possibility of continuous checking, and measuring a larger part of the surface than contact methods make possible. Their disadvantage is that the measurement of surface topography is indirect. This results in the need to compare experimentally obtained results from these devices with the values obtained by the contact profilometer [17].

Lu, Yi, Liu, Wang, and Ao [18] designed a similar method of measuring surface roughness to prove that the roughness measurement process is largely influenced by a laser light source that contains two colors—i.e., red, and green—creating a relationship between the overlap index and roughness.

According to C. Kang and H.X. Liu [19] and C. Lu [20], the surface profile and roughness of a product are crucial qualitative features that often indicate technical requirements for structural components. Ensuring the desired surface quality is essential for the proper functioning of the component. Moreover, the significance of the machined surface quality and the associated demands are continually rising [21,22].

The type and degree of roughness depend on the method of machining, the physical and mechanical properties of the material being machined, the cutting conditions, and, in particular, the size of the tool feed and the cutting speed [2]. The evaluated surface roughness parameters using laser profilometry were Ra, the mean arithmetic deviation of the profile, and Rz, the greatest height of the profile unevenness. The Ra and Rz parameters are the most monitored roughness parameters in practice and can provide sufficient information about the quality of the measured surface [23]. The feed rate of the cutting head is one of the most important and technologically most easily regulated technological parameters affecting the quality parameters of Ra and Rz [2].

**Figure 1:**
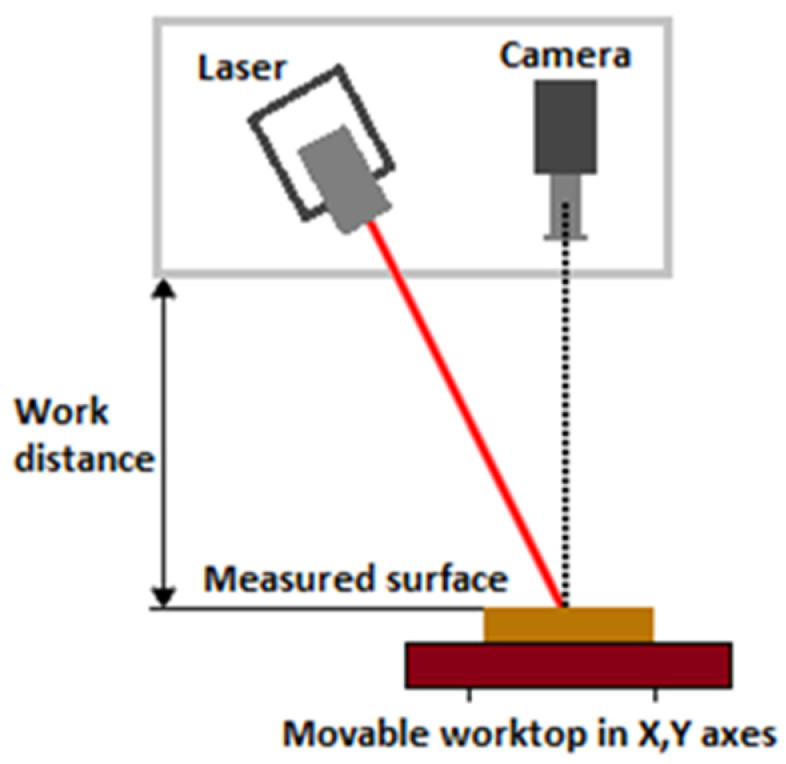
Principle of measuring with a laser profilometer.

Laser profilometry is a form of non-contact optical profilometric measurement of the surface of a scanned object, enabling a subsequent creation of a three-dimensional model of that object. The composition of such a profilometric system (Figure 1) is based on the presence of a laser beam emitted by an optical source of electromagnetic radiation and an image-reading device in the form of a camera, usually based on a CCD or CMOS sensing chip [12].

Laser profilometry can evaluate not only roughness parameters such as Ra and Rz, but also other surface characteristics such as Rp, Rq, and Rv. These parameters can provide additional information about the nature of individual parts of the surface. For example, Rp can indicate the height of the highest profile protrusion, Rq can give the average quadratic deviation of the profile, and Rv can reveal the depth of the largest depression in the profile. Therefore, using a laser profilometer for surface characterization can be beneficial in understanding the surface properties and optimizing the cutting process for specific materials [24].

The objective of the experiment is to compare different methods of measuring roughness on parting surfaces of samples from different materials. The samples included structural steel S235JRG1 and aluminum alloy AW 5754, which were cut using waterjet (AWJ) technology in a manufacturing plant.

Abrasive waterjet (AWJ) technology leaves visible scoring on the cut surface, which can significantly affect the dimensional accuracy of workpieces and the quality of the finished surface. The surface after cutting consists of two different regions: a smooth zone in the top part of the cut, which is the result of cutting wear, and a rough, grooved zone in the lower part of the cut, which arises because of deformational wear during water jet cutting [25,26,27]. A relatively smooth area, in the top part of the cut, is the result of the cutting wear zone, and the second grooved area, in the lower part of the cut, arises because of the deformational wear during waterjet cutting [25,26]. Most research works [28,29] qualify the surface condition through roughness parameters depending on the set cutting parameters. According to Chao, Zhou, Leu, and Geskin [27], the smooth region is characterized as homogeneous with a random profile and a slightly isotropic texture. The roughness parameters Ra and Rq are commonly used to quantify the surface condition, and they show a weak dependence on the cutting speed and are almost independent of the depth of cut for the smooth zone. However, for the grooved zone, the roughness characteristics—Ra and Rq—increase sharply with the increase in the depth of cut and the speed of the water jet. The findings suggest that the machined surface is smooth with inconspicuous grooves or waviness up to a certain depth of cut, while below this depth, the surface striation is pronounced, and the cut zone is visibly divided into two parts, smooth and grooved [28,30].

In many laboratory or industrial measurement applications, it is necessary to measure and evaluate the spatial shape of objects through non-contact 3D profile measurement. Laser profilometry is intended precisely for the quick and efficient solution of these tasks. The use of spatial control of the surface offers improvement of the properties of functional surfaces and progress in production processes, e.g., energy savings, etc. Therefore, it is in the interest of the technical development of production and control technologies to create all prerequisites for the comprehensive use of spatial surface assessment in normal production and metrological practice. For the reasons mentioned above, I focused the measurement experiment on the measurements of specific parameters of the roughness of the dividing surfaces and the analysis of the relationship between the material dividing speed and the type of material that is used most often in practice. To ensure accuracy, the roughness measurement experiment was performed using a non-contact laser profilometer, which was compared to a control roughness measurement using a contact roughness meter. The advantages of the spatial evaluation of the surface structure show that these are the metrological methods of the future. The evaluated spatial parameters of the 3D texture are based on a significantly larger amount of measured data and have a much higher reliability. The real image of the 3D surface obtained in this way contains a large amount of information that can be interpreted by an experienced observer. When using the spatial assessment of the surface structure, the dangerous neglect of the influence of some of the parameters from important surface properties is also significantly reduced. The processed data from individual experimental measurements were displayed in graphical dependencies and evaluated at the end of the paper. Classic contact measurement of roughness is still at the forefront in practice due to the standardized and clear surface roughness parameters. The practical management of the transition from 2D to 3D will represent a long-term process, both with the surface measurement itself, but also with the processing of the results and with the effective use of the obtained results. The success of enforcing the spatial quality control of the surface in metrological practice will undoubtedly be decided not only by technical but also by economic considerations. The practical benefit of the experiment is, based on the analysis of the sample surfaces, a view of whether the input parameters of the machine for cutting the material are set correctly. The comparison of different roughness measurement methods made it possible to determine their advantages and disadvantages, which are described in the final part of the paper. This information can be useful for determining the most suitable roughness measurement method for a particular type of material. Additionally, the results of the experiment can provide useful information for improving the water jet cutting process and optimizing the selection of cutting parameters for these materials.

## 2. Materials and Methods

The conducted experiments were aimed at monitoring the differences in the roughness of the cut surface of different types of materials produced under the same conditions of material separation, but at different feed rates of the cutting head adapted to the required resulting roughness and stiffness of the material. The tested samples were made of structural steel and aluminum alloy, and the roughness of the cut surface was measured in a non-contact and contact manner. When applying both methods, the cut areas measured were the same. The roughness parameters of the samples were measured and evaluated using both a contactless laser profilometer manufactured by KVANT and a contact roughness meter Surftest SJ-400 by the company Mitutoyo. The use of two different methods for roughness measurement allowed for a comparison of the results and helped to ensure the accuracy of the measurements.

**Figure 2:**
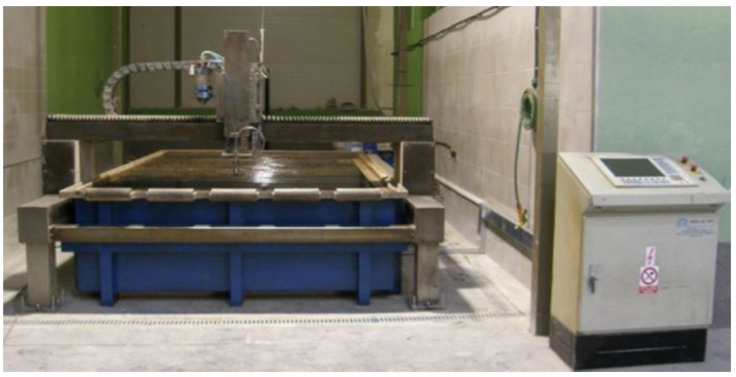
Cutting machine WJ4020 1Z-C0-P60 COBRA.

The actual production of samples consisted of cutting the material using the AWJ technology, which was done directly in the production plant of DRC s.r.o where the cutting machine WJ4020 1Z-C0-P60 COBRA (Figure 2) was used to separate the material, together with the high-pressure pump from FLOW SYSTEMS.

Table 1 is a technical specification table that shows the parameters of the cutting machine used to prepare the experimental samples.

These parameters play an important role in determining the quality of the cut surfaces and can also affect the roughness of the cut surfaces.

The analyzed materials (structural steel S235JRG1 and aluminum alloy AW 5754) were selected based on several factors, including their extensive usability, availability in the industry, and the variety of properties they exhibit.

Demonstration of the (nomenclature) marking:

- FS050—structural steel S235JRG1—cutting head feed rate (50 mm/min)
- FS100—structural steel S235JRG1—cutting head feed rate (100 mm/min)
- FS150—structural steel S235JRG1—cutting head feed rate (150 mm/min)
- AA120—aluminum alloy AW 5754—cutting head feed rate (120 mm/min)
- AA220—aluminum alloy AW 5754—cutting head feed rate (220 mm/min)
- AA370—aluminum alloy AW 5754—cutting head feed rate (370 mm/min)

**Figure 3:**
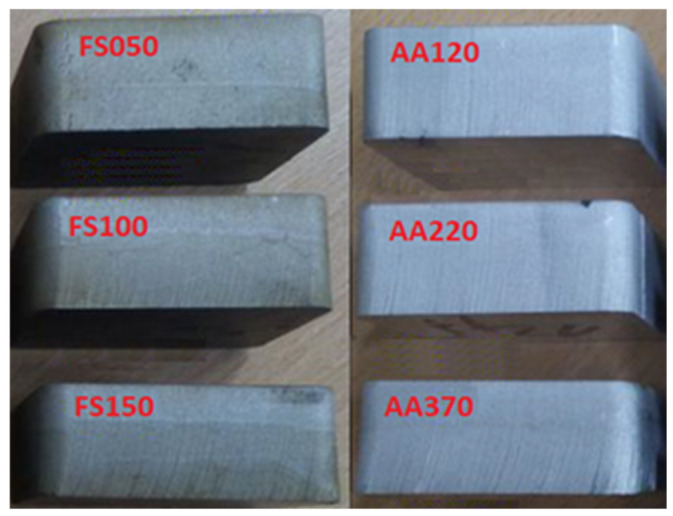
Marking of used samples from different materials.

Six samples were produced using the AWJ technology, as shown in Figure 3. Three of the samples were made of structural steel (FS050, FS100, FS150), and the other three were made of aluminum alloy (AA120, AA220, AA370). Each sample was produced using a different feed rate of the cutting head during the material separation process.

It is common for production companies to provide roughness specifications for parts or surfaces that they manufacture using various cutting or machining processes. These specifications provide customers with an indication of the quality of the finished product and help to ensure consistency in the manufacturing process. It is important to note, however, that the actual roughness values obtained during the manufacturing process can depend on a variety of factors the material properties of the workpiece and the accuracy of the cutting parameters used. The production company guarantees the roughness values (6.3; 12.5; 25 µm) for the cut surfaces only by changing the feed speed of the cutting head for different types of materials using the AWJ technology.

By providing roughness guarantees for parts produced using this technology, the production company can assure their customers of the quality of the finished product and demonstrate their commitment to meeting specific performance requirements.

Table 2 shows the input parameters set for cutting the material by the AWJ technology, constant for all samples produced as opposed to the changing speed of the cutting head.

**Figure 4:**
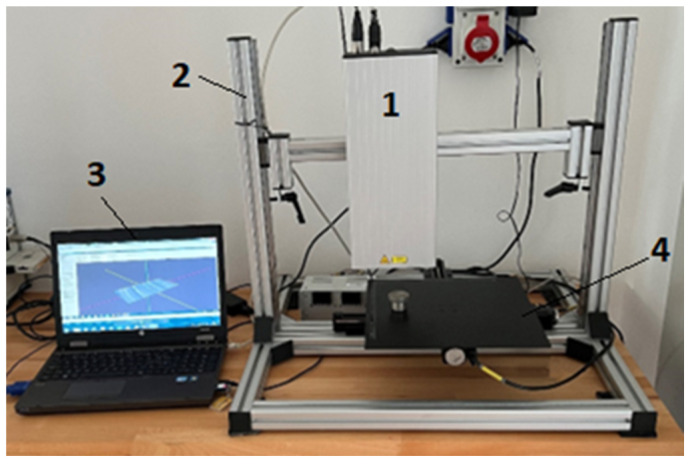
The laser profilometer setup. 1—Tube with laser and camera; 2—laser profilometer frame; 3—PC with operating and evaluation software; 4—worktop with step motors with movable in the X and Y axes.

The laser profilometer assembly is composed of both basic and additional parts, as shown in Figure 4. The basic parts consist of a sturdy aluminum support structure made up of components that allow for the vertical positioning of the measuring tube. The assembly also includes a worktop mounted on stepper motors that provide movement in the X and Y axes, a laser beam source, a lens, and a camera equipped with a CMOS (complementary metal oxide semiconductor) sensor and backlight. Additional parts that make up the assembly include a computer that runs the laser profilometer View control and evaluation software, as well as an image splitter.

The X- and *Y*-axis automated micrometric horizontal feed of the sample worktop was ensured by 2 Standa 8MT16-30 stepper motors with a maximum limit of up to 300 mm on each axis. The feed in the Z axis was resolved by the mechanical lifting of the entire profilometric tube using locking pins. The optical part of the laser profilometer system consists of an AVT Marlin 131B camera and a Tamron 23F50SP 50 mm lens with a visible area of 154 mm^2^ (22 × 7 mm). The frame design of the system allows the measurement of samples weighing up to a maximum of 8 kg with a positioning accuracy of 2.5 μm per step, with each step consisting of 8 μm. The camera sensor resolution is 0.02 mm/pixel.

When measured by the laser profilometer non-contact system, the experimental samples on the worktop were first placed in an anti-vibration mass. The first reason was that the machined surface of the sample was imperfectly fitted to the laser profilometer worktop and the sample on the laser profilometer worktop shifted during the movement of the stepper motors during the measurement. The second reason was to eliminate the influence of ambient vibrations, which can affect the quality of the measured data.

The experimental laser profilometer system allows for the measurement and evaluation of surface roughness parameters of various samples. The roughness parameters that can be measured using this system include Rq, Rv, Rz, Ra, and Rp. The roughness data collected from this system can be exported in CSV format, which is highly suitable for further processing of experiments in the form of raw data.

The Matrox Triple Head 2Go—Digital Edition image splitter ensures high-quality processing and display of images that include measured data. This device allows for the connection of up to three monitors to a single graphical output from a computer. In addition, by using the enhanced desktop option and an additional graphical output, up to four independent monitors can be obtained.

**Figure 5:**
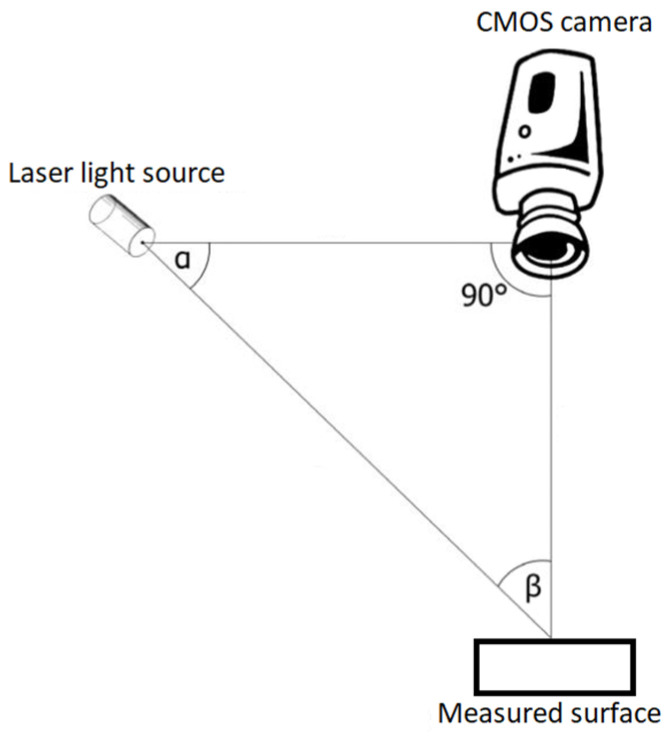
The principle of active triangulation.

The main principle of the laser profilometer system consists of using the well-known so-called triangulation principle, where the light source, together with the camera sensor and the examined object, forms the so-called triangulation triangle (Figure 5). A thin laser line is projected onto the measured profile; the image of the laser line is captured at an angle by a digital camera placed perpendicularly above the scanned surface; the profile of the object in the cross-section specified by the laser line is then evaluated from the scanned image. In this way, it is possible to create a real 3D profile from each scanned image. This contactless profilometer cooperates with a classic computer via the laser profilometer View profilometric software LPMView with the user environment. Laser profilometer View is used for communication with a linear laser profilometer, evaluating the data captured by the camera sensor, which is then processed in the desired form. FireWire [2] is required for communication.

**Figure 6:**
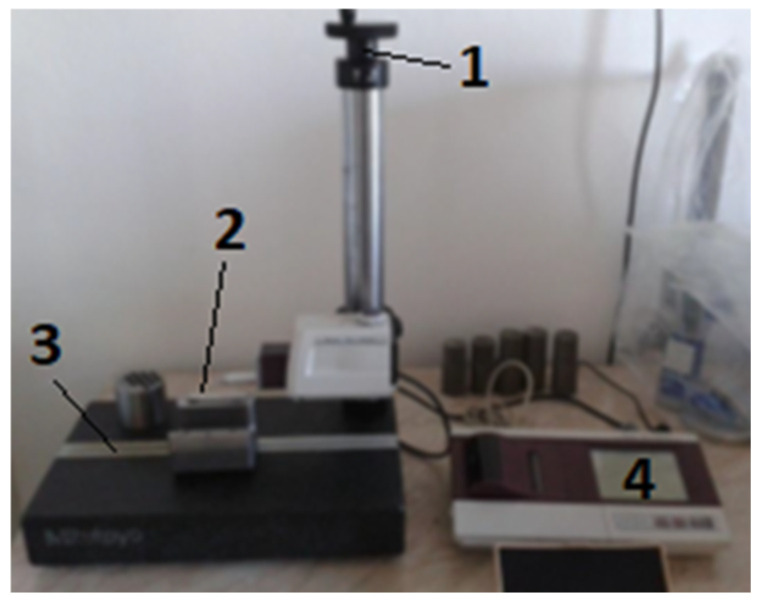
Surftest SJ-400 contact roughness meter. 1—Adjusting the standoff distance of the measuring tip; 2—measuring tip; 3—worktop; 4—touch display with the printer.

As mentioned, to verify the correctness and compare the measured values with the contactless laser profilometer, the measurement of samples was also carried out in a contact way, using the Mitutoyo Surftest SJ-400 roughness meter (Figure 6). Table 3 is the listed parameters of the contact roughness meter.

For each sample, the same section of the measured surface was chosen for the application of both measurement methods. The materials used for the samples were defined by the relevant standards as structural steel S235JRG1 and aluminum alloy EN AW 5754.

### Experimental Samples Measurement

Measurement of the roughness of the cut surfaces, created in the process of material separation by water jet technology (AWJ), was carried out on two types of materials. The measured samples were of the same thickness; however, they were made at different feed rates of the cutting head. Each material was used to create three samples.

**Figure 7:**
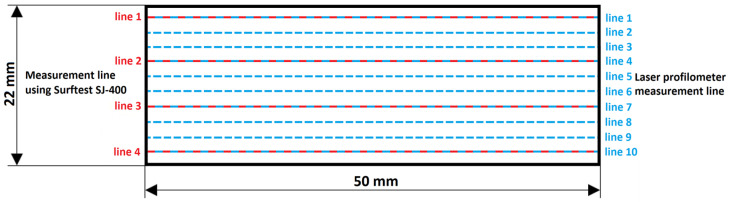
Lines of measurement are specified on individual samples.

The measurement of the roughness parameters Ra and Rz of the cut surface of the sample, when measured with the laser profilometer, was determined on each sample at ten lines of the cut surface measurement (Figure 7, right-hand side)—specifically, at the measurement lines 0.11; 2.31; 4.51; 6.71; 8.91; 11.11; 13.31; 15.51; 17.71; 19.91 mm from the bottom of the sample. The thickness of the sample material measured with both methods was the same.

Using the contact method—i.e., the Surftest SJ-400—the roughness parameters of the cut surface were determined in four measurement lines of the cut surface (Figure 7, left-hand side)—specifically, at the measurement lines of 0.11; 6.71; 13.31; 19.91 mm from the bottom of the sample. The number of measurements was reduced due to this method’s complexity and difficulty of measurement.

Table 4 displays the values measured with the Surftest SJ-400. These values serve as a comparison to the values measured with the laser profilometer and to verify the accuracy of the measurements.

## 3. Results and Discussion

When assessing the roughness of the surface, the Ra and Rz parameters were used, representing the arithmetic average roughness and the maximum height of the roughness profile, respectively. The surface of the samples was measured non-contacting in 181 steps with a step size of 0.11 mm at 10 measurement lines, with a step shift of 2 mm from the top of the sample. The Gain mode, which amplifies the image signal in the camera, was set to 1. The cutoff time, which determines the time for scanning the surface, was set to 19.520 ms. The input parameters of the laser profilometer were adjusted based on previous test measurements to provide the purest image preview and the least noise in the image.

In this section of the paper, the roughness data obtained from the surface of a sample that wares produced using the AWJ technology were evaluated using the laser profilometer. The data obtained for the sample material, varying with the feed rate of the cutting head, were plotted concerning the roughness parameters, Ra and Rz. The surface roughness was measured at ten different lines on three structural steel samples and three aluminum alloy samples. The input parameters used for setting up the laser profilometer to measure surface roughness are presented in Table 5. These parameters were consistent with those observed in previous studies by Ružbarský [2], Srivastava et al. [31], and Valicek et al. [32].

The measured surface roughness values of the individual samples were processed, resulting in the dependence of the mean arithmetic deviation of the Ra profile unevenness on the measurement line on the sample h at the feed rate of the cutting head FS050 mm/min, FS100 mm/min, FS150 mm/min, AA120 mm/min, AA220 mm/min, and AA370 mm/min, and the dependence of the greatest elevation of the unevenness of the Rz profile on the measurement line on the sample h at the feed rate of the cutting head FS050 mm/min, FS100 mm/min, FS150 mm/min, AA120 mm/min, AA220 mm/min, and AA370 mm/min. The results are also confirmed by the conclusions reached by Azhari et al. [33] and Jurko et al. [34].

**Figure 8:**
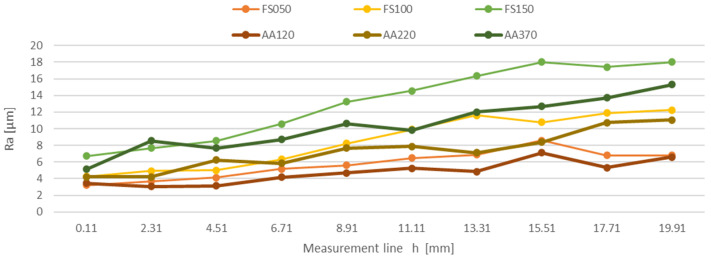
Plotted dependence of Ra on the measurement line for the materials FS050, FS100, FS150, and for the materials AA120, AA220, and AA370.

The plotted dependence of the parameter Ra (Figure 8) shows the surface roughness at the measurement lines of the sample at the feed rates of the cutting head 50, 100, and 150 mm/min for structural steel, and 120, 220, and 370 mm/min for aluminum alloy, respectively. These results are also confirmed by the conclusions reached by Azhari et al. [35] and Foldyna et al. [36].

**Figure 9:**
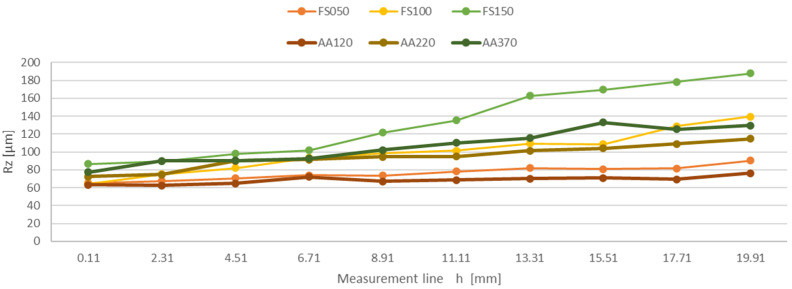
Plotted dependence of Rz on the measurement line for the materials FS050, FS100, FS150, and for the materials AA120, AA220, and AA370.

The plotted dependence of the Ra parameter (Figure 9) shows the surface roughness at the measurement lines of the sample at the feed rates of the cutting head 50, 100, and 150 mm/min for structural steel, and 120, 220, 370 mm/min for aluminum alloy. These results are also confirmed by the conclusions reached by Akkurt [37], Hreha et al. [38], and Boud et al. [39].

The curves of the monitored parameters Ra and Rz show an increasing trend on all samples depending on the line of measurement.

**Figure 10:**
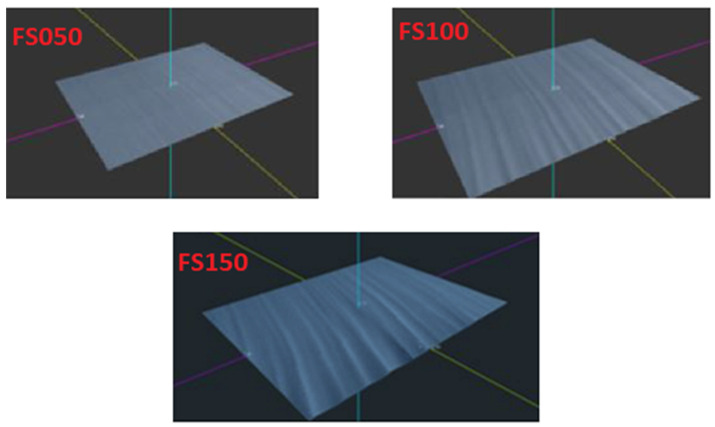
3D model of scanned S235JRG1 samples.

Figure 10 shows 3D models of scanned samples made of structural steel S235JRG1, which were created by placing a series of measured profiles side by side via a laser profilometer. The models show the 3D surface of the cut material of experimental samples captured by the laser profilometer. Figures show a clear impact of the cutting head speed on the surface quality. A greater difficulty of the cutting water jet passing through the material is visible on experimental samples (FS050, FS120, FS150), and the water jet left more depressions and unevenness on the surface of the cut areas. The comparison of the plotted dependencies of the parameters Ra and Rz (Figure 8 and Figure 9) for structural steel shows that the roughness values on the cut surface are more stepped, which reflects the greater strength and lower toughness of the evaluated material. The surface roughness of the cut areas in these experimental samples is greater across the entire cut surface than that of the aluminum alloy. These results are also confirmed by the conclusions reached by Tomas et al. [40], Kunaporn et al. [41], and Hascalik et al. [42].

**Figure 11:**
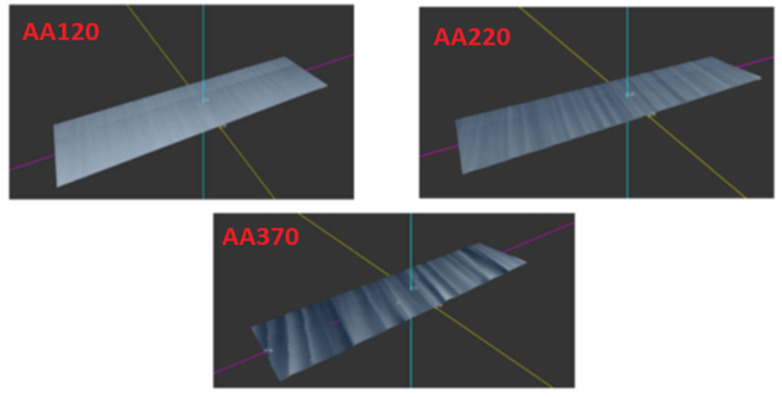
3D model of scanned EN 5754 samples.

Figure 11 shows 3D models of scanned samples made of AW 5754 aluminum alloy, which were created by placing a series of measured profiles side by side via a laser profilometer. The figures also show the effect of the cutting head speed on the surface quality of the aluminum alloy. A comparison of the created 3D models (Figure 10 and Figure 11) shows a clear difference in the surface quality of the samples of structural steel and aluminum alloy. Samples made of aluminum alloy (AA120, AA220, AA370) show a smoother cut across the entire profile of the cut surface, where the passage of the water jet through the material did not leave deep depressions on the surface of the cut areas, unlike on samples that were made of structural steel. The comparison of plotted dependencies of the parameters Ra and Rz (Figure 8 and Figure 9) for aluminum alloy shows that the roughness values of the cut surface are less stepped, which reflects the lower strength and greater toughness of the materials evaluated. These results are also confirmed by the conclusions reached by Akkurt et al. [43] and Monno et al. [44].

In the non-contact measurement method using laser profilometry, the surface area of the cut sample was divided into ten measurement lines, and for the contact measurement method using Surftest SJ-400, the surface area of the cut sample was divided into four measurement lines (Figure 7). To verify the correctness and compare the values measured with the laser profilometer, all samples were first measured with the contact Surftest SJ-400 roughness meter (Table 4). The mutual comparison of the measured data shows that the values of the Ra parameter measured on the laser profilometer are within the range of values that were measured with the contact Surftest SJ-400 roughness meter. The values of the Rz parameter that were measured with the contact Surftest SJ-400 roughness meter are two-to-three times smaller than the values measured with the laser profilometer. Similar values and conclusions were reached by the authors Krenicky [1] and Hlavac et al. [11]. Krenicky [1], Mital et al. [17], and Ružbarský [45] assumed the reason might be that the contact roughness meter’s tip diameter could not enter every surface recess (the top of the measuring tip is of a diameter larger than some surface recesses) and partly due to the image noise during measurements with the laser profilometer, where in some measurement lines there was a reflection of laser light from the surface of the sample to the CMOS camera, which resulted in a sharp increase in Rz values and thus a measurement error. This measurement error can be corrected by removing such distorted values from the exported data.

## 4. Conclusions

Roughness control using a laser profilometer can provide valuable information about the surface quality of the material after the cutting process. The conducted experiments suggest that in praxis, crucial factors that affect the surface roughness parameters Ra and Rz is the AWJ cutting technology, the feed rate of the cutting head, and the type of material. The results of the experiment can be summarized in the following points:

- All measured dependencies show an increasing trend of the selected roughness parameter values, and the surface roughness varies linearly, with the depth of the cut done by the AWJ cutting technology.
- The smooth zone occurs mainly in the first part of the cut of the separated area. This is also confirmed by the plotted dependencies Ra and Rz, where we measured the smallest initial surface roughness.
- In the smooth zone of the separated surface, the measured values of the Ra and Rz parameters are closest to each other, but even at these values, there is an obvious difference in the roughness of the surface of the cut area depending on the speed of the cutting head.
- With a decrease in the feed rate of the cutting head, the size of the smooth zone (characterized by lower roughness parameters) increases.
- The linearly increasing roughness of the cut surfaces applies to the smooth and medium-smooth zones of the cut surfaces. An exponential increase in the roughness of the cut surfaces occurs in the middle and end zones of the cut surfaces.
- The occurrence of visible reliefs on the cut surfaces was less common in aluminum samples than in structural steel samples. It follows from the above that to achieve the same surface roughness in different materials, a different cutting head speed must be used.
- This non-contact method of measuring surface roughness enables checks in real-time, which is a very important advantage in terms of overall production efficiency.

The results of the experiment indicated that the surface roughness of the samples produced by the DRC company did not exceed the declared roughness of any of the samples used—i.e., the input parameters of the material cutting machine are set correctly. However, the Surftest SJ-400 contact roughness meter used to measure the roughness parameters did not provide a complete picture of the roughness on the surface being assessed or the roughness in different parts of the surface under evaluation.

To summarize, the laser profilometer provides a set of values that describe the surface areas at each step of measurement, allowing for the evaluation of multiple parameters and creating a 3D image of the surface being measured for better visualization of the surface characteristics. Overall, the experiment provides valuable insights into the comparison of different roughness measurement methods and their suitability for specific applications.

A certain disadvantage is glossy or highly reflective surfaces, which can cause issues when measuring surface roughness parameters using contact-free methods. The laser light can reflect off the surface, creating unwanted reflections that may be captured by the CMOS camera and these reflections can lead to inaccurate measurements of surface roughness parameters. In such cases, contact methods may be more appropriate for obtaining accurate measurements. In summary, while contact-free methods, such as laser profilometry, can provide accurate measurements of surface roughness parameters on many surfaces, highly reflective surfaces can pose challenges.

## Figures and Tables

**Table 1:** Specification of the cutting machine WJ4020 1Z-C0-P60 COBRA.

Parameter	Value
Working speed (max)	20,000 mm/min
Accuracy	+/−0.04 mm/300 mm
Repeatable accuracy	+/−0.03 mm
Speed (max)	30,000 mm/min
Movement in the *Z*-axis	500–700 mm
Cutting heads (No)	1–4
Portal width (max)	4 m
Length of linear guide (max)	30,000 mm

**Table 2:** Input parameter setting of the material cutting machine using the AWJ technology.

Parameter	Value
Type of abrasive	Australian Grenade
Amount of abrasive (g/min)	430
Diameter of the guide tube (mm)	0.889
Diameter of the water jet (mm)	0.406
Abrasive grain size	MESH 80
Pressure (MPa)	379.21

**Table 3:** Technical parameters of the Surftest SJ-400 roughness meter.

Parameters	Value
Speed of return (mm/s)	0.5; 1.0; 2.0
Positioning	±1.5° (inclination); 10 mm (up/down)
Evaluated parameters	0.889
Measuring speed (mm/s)	0.05; 0.1; 0.5; 1.0
Measurement range/resolution (µm)	P (primary); R (roughness); W (filtered waviness)
Cutoff length (mm)	0.08; 0.25; 1.8; 2.5; 8

**Table 4:** Measured surface roughness parameters of the cut surface using Surftest SJ-400 in individual measurement lines.

Declared Roughness by the Company	Measurement Line	Structural Steel S235JRG1		Aluminum Alloy AW 5754	
		Ra [µm]	Rz [µm]	Ra [µm]	Rz [µm]
6.3	1	2.61	16.4	2.18	13.9
	2	2.88	15.5	3.35	16.2
	3	3.95	17.5	4.12	17.2
	4	4.36	19.5	5.74	25.8
12.5	1	2.53	14.7	3.59	18.9
	2	3.32	20.5	5.27	23.9
	3	5.96	33.2	8.71	39.2
	4	9.42	42.2	9.38	41.2
25	1	3.45	19.4	6.25	20.7
	2	4.25	28.3	8.72	41.5
	3	10.89	56.4	14.12	64.9
	4	15.73	59.4	21.34	74.8

**Table 5:** Input Parameters for Sample Measurement with Laser Profilometer Setting.

Scan Distance—Axis Y	Scan Line—Axis X
Scan lines count 200 µm	4000 µm
Scan lines step 110 µm	

## Data Availability

Not applicable.
